# Enhanced electrochemical performance of a polyaniline-based supercapacitor by a bicontinuous microemulsion nanoreactor approach†

**DOI:** 10.1039/d4ra07348g

**Published:** 2025-01-14

**Authors:** Yelriza Yeszhan, Sagydat Duisenbekov, Dana Kurmangaliyeva, Dana Kazhigitova, Perizat Askar, Yerbol Tileuberdi, Aishuak Konarov, Salimgerey Adilov, Nurxat Nuraje

**Affiliations:** a Department of Chemical and Materials Engineering, School of Engineering & Digital Science, Nazarbayev University Astana 010000 Kazakhstan nurxat.nuraje@nu.edu.kz; b Department of Chemistry and Chemical Technology, Al-Farabi Kazakh National University Almaty 050040 Kazakhstan; c Institute of Natural Sciences and Geography, Abai Kazakh National Pedagogical University 13, Dostyk ave. Almaty 050010 Kazakhstan; d Department of Chemistry, School of Sciences and Humanities, Nazarbayev University Astana 010000 Kazakhstan sadilov@nu.edu.kz; e Lab of Renewable Energy, National Laboratory Astana, Nazarbayev University Astana 010000 Kazakhstan

## Abstract

Polyaniline (PANI)-based supercapacitors suffer from environmental and mechanical instabilities. In this work, a novel bicontinuous microemulsion approach was developed to fabricate a unique nanofibre structure of polyaniline and its 3D-crosslinked network using crosslinking chemistry, which improved both the mechanical and electrochemical performance of a PANI-based supercapacitor. The polyaniline nanofibers and its 3D-crosslinked networks produced by bicontinuous nanoreactors were investigated using experimental tools, such as SEM, FTIR, BET, TGA and DSC. Electrochemical evaluations for the above polyaniline nanofibers and its 3D-crosslinked materials was performed *via* cyclic voltammetry and galvanostatic charge–discharge measurements. The result of this study demonstrated that the PANI nanofiber exhibited the highest specific capacitance of 280.4 F g^−1^ at a current density of 1 A g^−1^, while both PANI-based supercapacitors made of nanofibers and 3D-crosslinked materials retained good cycling stability of 98% during continuous redox cycling.

## Abstract 2

A novel bicontinuous microemulsion nanoreactor was developed to produce unique PANI nanofibers and a 3D crosslinked PANI architecture to enhance the mechanical stability and electrochemical performance for further application in supercapacitors.

## Graphical abstract

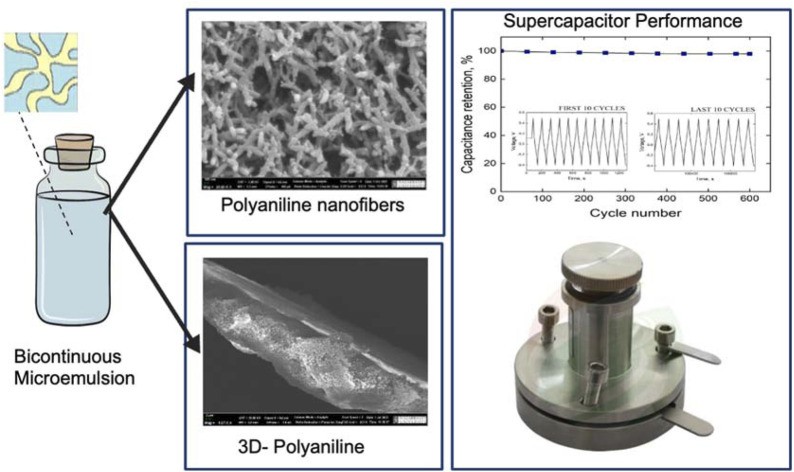

## 1 Introduction

Supercapacitors have demonstrated their potential applications in the fields of portable electronics, electric vehicles, and renewable energy systems as they possess high power density, and they can also be combined with batteries that have high energy density for various applications.^1^ Although a variety of materials have been explored as electrode materials for supercapacitors,^2,3^ polyaniline (PANI) is promising with its exceptional properties, such as high conductivity, light-weight and ease of synthesis.^4^ However, to fully realize the utilization of PANI and other conducting polymers in supercapacitors, there are still scientific challenges that need to be resolved.^5^

Polyaniline, a conducting polymer, can be used as an electrode of supercapacitors as it has multiple oxidation states.^6^ The emeraldine salt form is the most conductive and thus is suitable for use in supercapacitors. The electrochemical performance of PANI-based supercapacitors is largely owing to the faradaic redox reactions that occur within the polymer, which results in high specific capacitance and energy density. In addition, it possesses excellent electrochemical properties, is easy to synthesize, and stable in environmental conditions, which enables its use as an electrode in supercapacitors.^7,8^

However, certain challenges in the practical application of PANI are listed below: (1) mechanical degradation during charge–discharge cycles can cause loss of electrical contact; (2) limited cycling stability of conducting polymers during repeated redox cycling; and (3) low conductivity in oxidized states in some cases, reducing charge transfer efficiency and overall performance.^9^

To overcome the above challenges, scientists have developed different types of strategies, which include crosslinking, doping, functionalization,^10,11^ morphology selection^12^ and composite formation.^13^ Furthermore, formation of PANI composites with other materials, such as carbon nanotubes,^14^ graphene,^15^ or metal oxides,^16^ improved the supercapacitor performance *via* enhancing the mechanical and conductivity properties. Cross-linking PANI with other polymers or small molecules^17,18^ mitigated the mechanical degradation and enhanced cycling stability. Doping PANI with various dopants^19^ increased its conductivity and electrochemical properties, which leads to better performance and higher stability during cycling. Although the above strategies have been employed, there are still challenges that exist in intrinsic polyaniline-based supercapacitor studies.

**Fig. 1:**
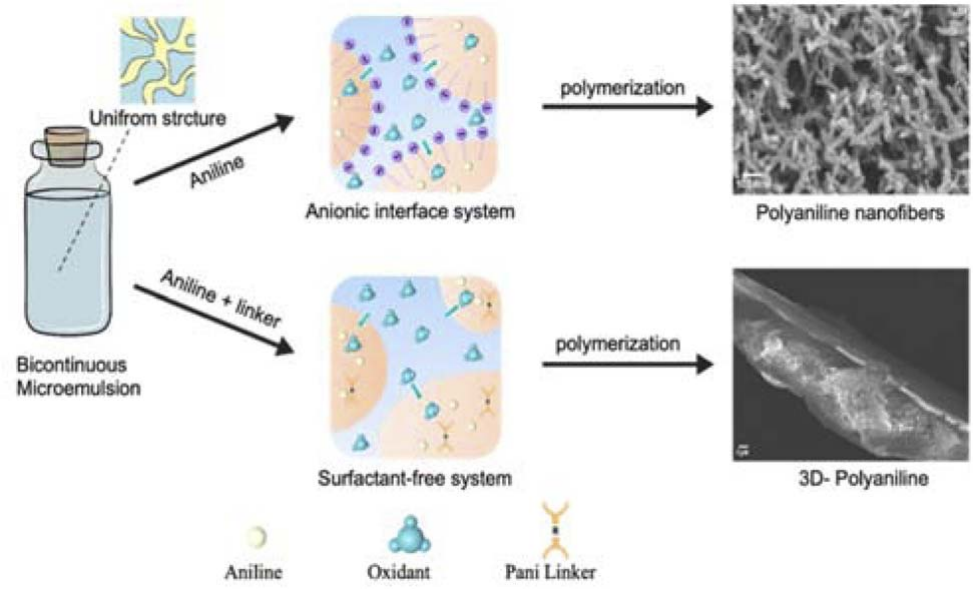
Schematic of synthesis of PANI nanofibers and 3D PANI *via* the microemulsion method.

To overcome these limitations, this study applies, for the first time, a bicontinuous microemulsion polymerization method to address the mechanical degradation, cycling stability, and charge transfer efficiency of PANI-based supercapacitors.^20^ This technique enables the synthesis of unique nanostructures of PANI, combining doping and crosslinking to enhance the performance. The bicontinuous microemulsion polymerization method is particularly important because it facilitates the formation of highly controlled nanostructures and three-dimensional networks, which are essential for improving both mechanical and electrochemical properties. As shown in Fig. 1, this method provides a versatile platform for tailoring the morphology of PANI and optimizing its performance under different doping levels, paving the way for more efficient and stable supercapacitor applications.

## 2 Results and discussion

In this research, for the improvement of the intrinsic polyaniline supercapacitor performance, the unique strategy we adopted is to enhance charge transfer efficiency and redox cycling stability *via* designing nanofiber structures of polyaniline and employing crosslinking chemistry for the fabrication of a mechanically stable 3D polyaniline freestanding electrode system. Therefore, unique bicontinuous microemulsion nanoreactor systems were first applied to produce nanofiber structures of polyaniline and fabricate crosslinked 3D-polyaniline freestanding films *via* the design of a unique PANI crosslinker.

### 2.1. Fabrication of PANI nanostructures and its 3D crosslinked films by a bicontinuous nanoreactor

As described in the Experimental section, two different types of bicontinuous systems (anionic bicontinuous and surfactant free bicontinuous) were selected to fabricate PANI nanofibres and 3D-crosslinked materials, respectively. The anionic bicontinuous microemulsion consisting of SDS surfactant, cyclohexane and water was applied as a nanoreactor to fabricate nanofibers of polyaniline. To fabricate 3D-crosslinked polyaniline networks, a surfactant free bicontinuous system was selected to avoid the influence of surfactants on the crosslinking of polyaniline. To produce a nicely tailored 3D structure ratio of linker to monomer 1 : 100, 1 : 150 and 1 : 200 were tested. However, the conductive form of 3D crosslinked PANI was possible to obtain with only the ratio of 1 : 200.

The Fourier transform infrared (FTIR) spectra of PANI nanofibers and 3D PANI are shown in Fig. S3.† Distinct absorption peaks are observed at around 770 cm^−1^ (representing aromatic ring and out-of-plane deformation vibrations), 1480 cm^−1^ (indicating benzenoid ring-stretching vibration), and 1560 cm^−1^ (corresponding to quinonoid ring-stretching vibrations). These absorption bands are characteristic of PANI.^21^ The band observed at around 1240 cm^−1^ is associated with the stretching of the C–N^+^ polaron structure, which supports the presence of doping in the PANI films.^22,23^ The presence of an ether group (C–O–C) (1255–1032 cm^−1^) is observed in the linker structure of the 3D PANI.^24^

The TGA and DSC curves of polyaniline nanofibers and crosslinked polyaniline are compared in Fig. S4.† The TGA curve shows a weight decrease at temperatures up to approximately 100 °C, which is caused by the evaporation of water and volatile substances adsorbed.^12^ Within the temperature range of 100 °C to 300 °C, there is a steady decrease in weight, which is most likely caused by the thermal breakdown of the polymer's side chains and partial degradation of the polyaniline backbone. The weight loss seen between 300 °C and 500 °C indicates a substantial disintegration of the polyaniline nanofibers. The weight decreases to around 20% by 600 °C, suggesting the production of a constant carbon residue.^25,26^ The DSC curve exhibits multiple thermal transitions, beginning with an endothermic peak at around 50 °C, which is associated with the evaporation of moisture. Within the temperature range of 100 °C to 300 °C, there is a wide endothermic area that indicates the ongoing absorption of energy as the polymer begins to degrade. A thermal degradation occurs within the temperature range of 300 °C to 500 °C, signifying the occurrence of oxidative degradation and crosslinking processes. At temperatures beyond 500 °C, there is a significant rise in heat transfer, indicating the breakdown of any residual polymer portions and the onset of burning. In comparison to crosslinked polyaniline, the nanofibers demonstrate less thermal stability, as they begin to decompose noticeably at approximately 300 °C. However, they exhibit a higher temperature at which carbon formation occurs. These changes in thermal behaviour can be attributed to the lack of cross-linking.^27^

**Fig. 2:**
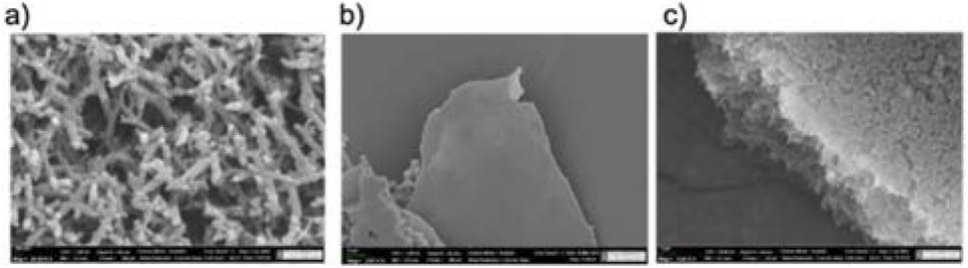
Surface morphology of (a) PANI nanofiber, (b) 2D and (c) 3D PANI.

**Fig. 3:**
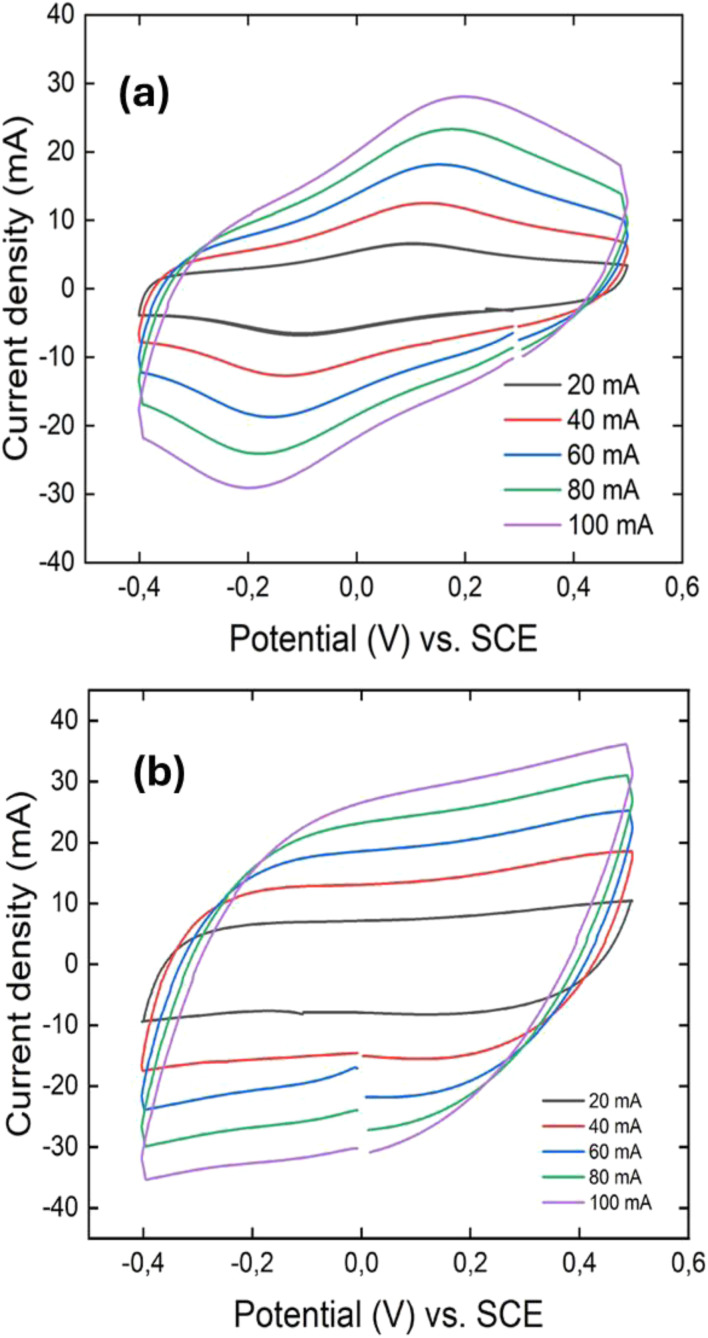
CV curves of PANI fiber (a), and 3D PANI (b).

The surface morphologies of PANI nanofibers, a 2D film, and 3D crosslinked PANI were explored using a Scanning Electron Microscope (SEM) as shown in Fig. 2. Fig. 2a demonstrates the surface of nanofibers, which is highly gritty and rough in appearance. The nanofibers have an average diameter of 130 nm and are consistent over the entire sample, as the figure demonstrates. The crystallization of oxidized organic molecules might be responsible for this phenomenon. A thin polyaniline film was produced as a result of polymerization carried out on an ice template. The coating is extremely porous and relatively thin, as can be seen in Fig. 2b. This structure is due to the high concentration of the oxidant, which causes the creation of a large number of nucleation centers. In Fig. 2c and 3D polyaniline has a loose, porous structure like a sponge. Nanoparticles are short and fibrous. Between the fibers, the pores formed as a result of tailoring with the linker. However, it was difficult to determine the distinct particle size in the 3D-crosslinked PANI materials by magnifying the SEM image due to the low electrical conductivity of the polymer.

Taking into account that electrochemical supercapacitors include electron and ion transport pathways at the interface between the active material and the electrolyte, the surface area of the synthesized materials was explored by Brunauer–Emmett–Teller (BET) N_2_ adsorption isotherms, as shown in Fig. S5.† According to BET measurement, the specific surface areas of the materials are 30 m^2^ g^−1^, 10 m^2^ g^−1^, and 27.7 m^2^ g^−1^ for PANI fiber, 2D PANI film and 3D PANI, respectively. The three different PANI nanostructures show relatively low and similar surface areas.

### 2.2. Electrochemical performance of PANI nanofibres and its 3D-crosslinked networks

A preliminary investigation on the electrochemical behaviour of the synthesised materials was performed using a symmetric two-electrode split cell based on the hypothesis that the nanofiber structure and crosslinking can improve the supercapacitive performance. Both cyclic voltammetry (CV) and galvanostatic charge–discharge (GCD) were used to study the capacitive behaviour of the above synthesized materials. However, for the PANI film obtained by the ICE-templated approach, it is difficult to obtain a stable measurement, which may be ascribed to the delicateness and non-uniformity of the PANI film. For CV and GCD measurements, a −0.5 V to 0.5 V voltage window and a current density of 1 A g^−1^ were used.

CVs at different scan rates for the PANI nanofiber and crosslinked 3D PANI are depicted in Fig. 3.

As illustrated in Fig. 3a, the CV curve of PANI nanofibers exhibits a quasi-rectangular shape with a single pair of redox peaks, indicating a combination of capacitive behavior and pseudocapacitance. The quasi-rectangular background reflects electric double-layer capacitance, while the prominent anodic (oxidation) and cathodic (reduction) peaks correspond to the leucoemeraldine ↔ emeraldine redox transition, the dominant charge storage mechanism under the given conditions. This observation deviates from the typical CV profile of PANI, which usually shows two distinct redox pairs: leucoemeraldine ↔ emeraldine and emeraldine ↔ pernigraniline.^8^ The absence of the second redox peak can be explained by several factors. Firstly, the nanostructured morphology of the PANI nanofibers, synthesized *via* a microemulsion method, results in a relatively high surface area (30 m^2^ g^−1^) and roughness,^28^ as confirmed by SEM and BET. These features enhance the kinetics of the first redox process, making it more pronounced, while the second process (emeraldine ↔ pernigraniline) is suppressed due to the inherent instability and lower conductivity of the pernigraniline state. Secondly, the mildly acidic H_3_PO_4_/PVA-based polymer gel electrolyte stabilizes the emeraldine state but disfavors the transition to the pernigraniline state.

Fig. 3b indicates that the cyclic voltammetry curve of 3D crosslinked PANI has a rectangular shape, which indicates the pseudocapacitive behaviour of the material, where no redox peaks are observed. This phenomenon is associated with a higher degree of disorder compared to the PANI fiber, which prevents the formation of stable redox states of the polymer. The rectangular shape of the curve is related to the more active sites for electrochemical reactions provided by the 3D network of PANI.^29^ Even though the material has higher conductivity, its small surface area limits the amount of charge that can be stored through redox reactions that happen on the surface.^30^

**Fig. 4:**
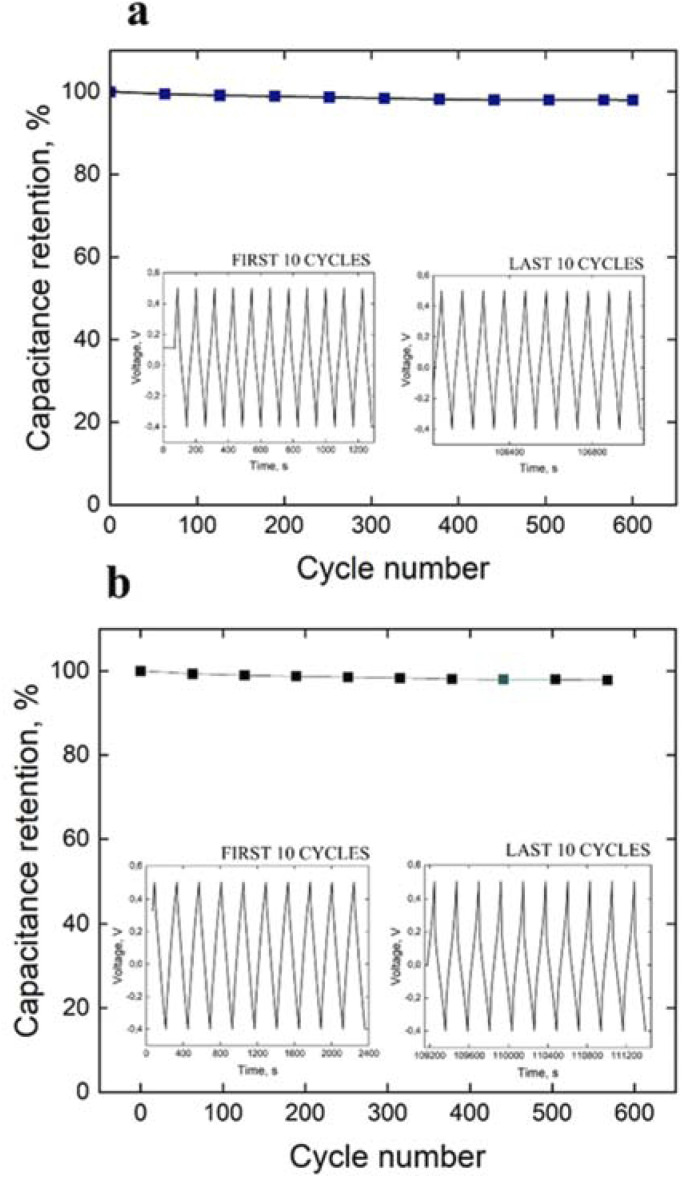
Cycle life test and GCD curve of the first and last 10 cycles of (a) PANI fiber and (b) 3D crosslinked PANI at a current density of 1 A g^−1^.

Fig. 4 displays the GCD curve and cycle life of PANI fiber and 3D crosslinked PANI at a current density of 1 A g^−1^, respectively.

From the GCD curve, the specific capacitance of the electrodes was calculated using eqn (1):1
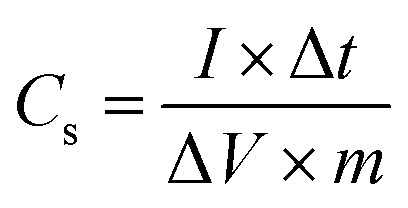
where *I* is the constant current in *A* applied in the charge–discharge process, Δ*t* is the discharge time in s, *m* is the mass of the active material in g, and Δ*V* is the voltage window in charge–discharge experiments in V.^8^

According to the specific capacitance equation calculated from the charge–discharge curve, the specific capacitance values of PANI fibers and 3D crosslinked PANI networks at a current density of 1 A g^−1^ are 280.4 F g^−1^ and 58.8 F g^−1^, respectively.

Even though the specific surface areas of both materials are similar, the specific capacitance values are significantly different. This indicates that specific surface area alone is not the sole determinant of capacitance; the morphology, porosity, and structural organization of the material also play pivotal roles.

The significantly higher capacitance of PANI nanofibers can be attributed to their gritty, rough surface morphology and their highly fibrous structure, which provides abundant electroactive sites for ion adsorption and charge transfer.^28^

The low specific capacitance of 3D PANI is due to its relatively small surface area (Fig. S5†) and low electrical conductivity.

Moreover, using eqn (2) and (3), the energy and power density of the cells are calculated.2
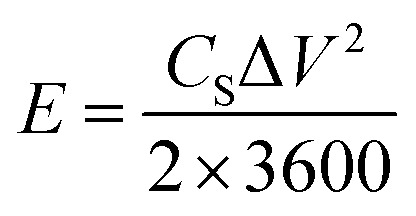
3
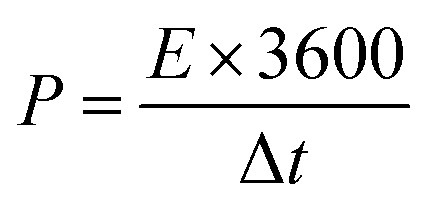
where *I* is the constant current in A applied in the charge–discharge process, Δ*t* is the discharge time in s, *m* is the mass of the active material in g, and Δ*V* is the voltage window in charge–discharge experiments in V. Energy and power densities of the polymer-based supercapacitors are 14.02 W kg^−1^ and 900 W kg^−1^ for PANI fiber and 2.9 W kg^−1^ and 183 W kg^−1^ for 3D PANI, respectively. Although these values are lower than, but comparable to literature values, it showed very good (98%) cyclability rate.^28^

Fig. 4 also summarises the specific capacitance of assembled symmetric cells of PANI fiber and 3D crosslinked PANI networks. As distinguished from the charge–discharge curves, PANI fiber has 1000 cycles in 30 hours, while 3D PANI has only 637 cycles in 41 hours, which indicates that the first material has longer cycle life, while the second one has a higher cycle rate. Moreover, both supercapacitors lost their capacitance in the first 200 cycles. This phenomenon can be associated with the initial surface activation, and the first cycles are required to activate the electrode surface and establish a stable electrochemical interface between the electrode and the electrolyte. Once the surface is fully activated, the electrodes stabilise. Nevertheless, during activation, only 2% of the capacity is lost, and throughout the entire process, both supercapacitors retain 98% of the initial capacity.

## 3 Experimental

### 3.1. Materials and reagents

Aniline (99.5%), cyclohexane (99%), sodium dodecylsulfate (SDS) (99%), pentanol (98%), hydrochloric acid (37%), ammonium persulfate (98.0%), sodium carboxymethyl cellulose (CMC), and benzene (98%) were purchased from Sigma-Aldrich. The stainless-steel mesh used as the current collector was carefully cleaned with acetone and ethanol to remove any impurities.

### 3.2. Synthesis of the crosslinking agent

**Fig. 5:**
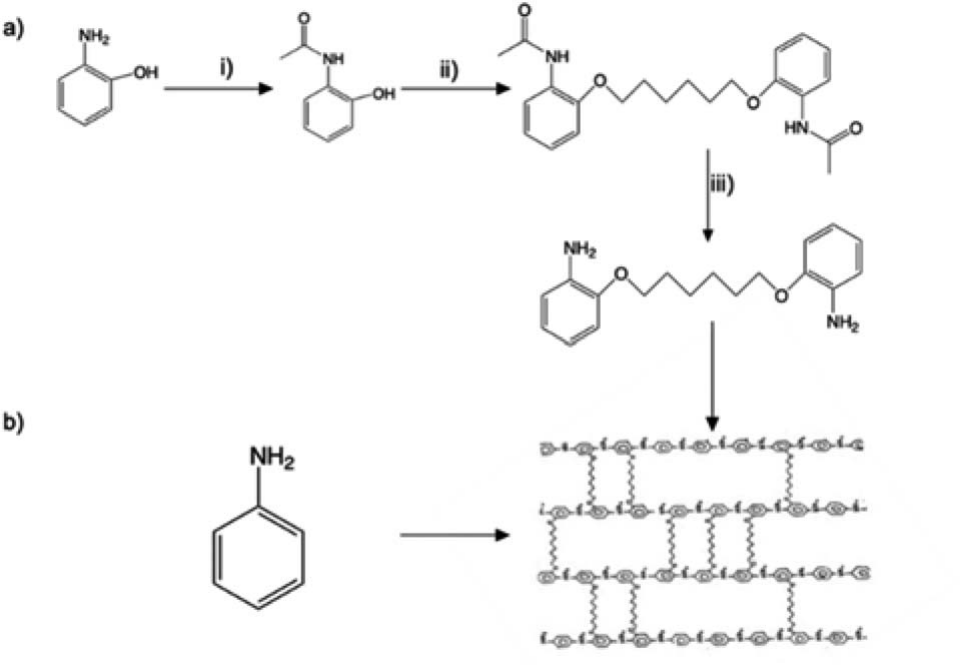
Chemical synthesis of crosslinked 3D PA. (a) Synthesis of the linker monomer; (b) 3D PA synthesis reaction.

Synthesis of the crosslinking agent, 2,2′-(hexane-1,6-diylbis(oxy))dianiline, was performed by the three steps in Fig. 5a, which are described in detail as follows.

(i) Synthesis of *N*-(2-hydroxyphenyl) acetamide: 2-aminophenol (8.34 g, 76 mmol) and THF (60 ml) were mixed together, and then, 8 ml of acetic anhydride was added drop by drop until a light brown solid formed. The temperature was kept below 50 °C, and the mixture was stirred continuously. The mixture, after being cooled to room temperature, was mixed with 50 ml of hexane and continuously stirred for 1 hour to obtain a precipitate. After that, the resulting product was filtered and washed with hexane (50 ml × 2). The solid was left overnight to dry, resulting in a white, crystalline powder.

(ii) Synthesis of 2-acetyl aminophenol. *N*-(2-Hydroxyphenyl) acetamide (5 g, 33.1 mmol) and anhydrous K_2_CO_3_ (6.862 g, 49.65 mmol) were put into a round-bottom flask containing 100 ml of acetonitrile and stirred for 30 minutes at room temperature. Then dialkylhalide with a carbon length of C6 (0.5 mmol eq.) was added dropwise to the mixture and refluxed for 16 hours. After the reaction was finished, the solvent was removed by evaporating it at a lower pressure while also being washed out with DI water. The sediment was named compound-2 and left overnight.

(iii) Synthesis of 2-alkoxybenzenamine. Compound-2 (9.1 g, 11 g, and 5 g) was dissolved in 40 ml ethanol, and a solution of KOH (16 g, 26 g, and 15 g) in 60–75 ml ethanol was added, continuously stirred, and heated at 90 °C for 21 h. After that, the solvent was evaporated under reduced pressure and diluted with water. The product was then extracted from an aqueous layer with DCM (100 ml × 2). The combined organic layers were dried with MgSO_4_. Finally, the solvent was evaporated under reduced pressure to obtain the final product.

2,2′-(Hexane-1,6-diylbis(oxy))dianiline: the crosslinker molecule was characterized by NMR spectroscopy. Fig. S1 and S2,† respectively, show ^1^H NMR and ^13^C NMR spectra and the corresponding peaks assigned below confirmed the chemical structure of the crosslinker: ^1^H NMR (CDCl_3_, 500 MHz): *δ* (ppm) 1.56 (bs, 4H), 1.85 (t, 4H), 3.78 (s, 4H), 4.00 (t, 4H), 6.79 (m, 4H),6.85 (m 4H); ^13^C NMR (CDCl_3_, 125 MHz): *δ* (ppm) 26, 29, 68, 112, 115, 118, 122, 135, 147.

### 3.3. Polyaniline (PANI) nanofiber synthesis

An anionic bicontinuous microemulsion system consisting of cyclohexane, SDS + *n*-pentanol and water was used as a nanoreactor to synthesize PANI nanofibers.^31^ To produce the PANI nanofibers, 0.658 ml aniline monomer was dissolved in 4.61 ml cyclohexane and this solution was named A. Then 1.5 g of SDS was mixed with 2.764 ml *n*-pentanol, and this mixture was added to Solution A and stirred for 10 min to get a cloudy dispersion. Finally, 7.5 ml ammonium persulfate (APS) solution (0.25 mol) prepared in 1 M HCl was added to the monomer-containing solution, poured into a vial and left for 24 hours for complete polymerization. The obtained green powder was several times washed with ethanol and DI water to remove impurities.

### 3.4. Synthesis of a 2D polyaniline (PANI) film

Chemical oxidative polymerization on ice at 0 °C was used to create a 2D PANI film utilizing aniline and APS as the starting materials. The water in the Petri dish was frozen at a temperature of −20 °C, and the dish was flipped over to achieve a flat surface. The molar ratio of aniline to APS was 8 : 3; therefore, 0.25 M aniline in 1 M hydrochloric acid and 0.25 M APS in 1 M hydrochloric acid were each added onto the ice surface in turn. The development of two-dimensional nanosheets with a diameter of several millimetres was apparent to the naked eye after the reaction had taken place for three minutes. Following the step of transferring the PANI film to a Petri dish, the resulting polymer was subjected to several washes with deionized water and then dried in a vacuum oven for seven days.^32^

### 3.5. Synthesis of 3D polyaniline (PANI)

3D PANI was synthesized using a bicontinuous microregion of a surfactant-free microemulsion system of benzene, ethanol and water.^33^ In a typical synthesis of the 3D PANI system, a previously synthesized LC6 linker (which followed the synthesis procedure shown in Fig. 5a) was mixed with aniline in a ratio of 1 : 200, and the resulting mixture was dissolved in benzene in a weight ratio of 20 : 80%. After that, APS was dissolved in 1 M hydrochloric acid at a concentration of 5 mg ml^−1^. Then, the calculated amount of components was gently mixed and left for 12 hours to complete polymerization (Fig. 5b). To eliminate contaminants, the obtained product was rinsed numerous times with ethanol and deionized water.

### 3.6. Electrochemical measurement

0.19 g of polymeric materials were mixed with 0.1 g of CMC as a binder to make an electrode. DI water was added to the resulting mixture to obtain a suspension. The slurry was transferred to the stainless-steel mesh using the blade casting method. The prepared electrodes were named PANI fiber and 3D PANI and placed in a vacuum oven at 60 °C for 4 hours. All the prepared electrodes had a thickness of 0.15 μm. To assemble the supercapacitor, the prepared electrodes were cut into 16 mm diameter circles, and a H_3_PO_4_/PVA-based polymer gel electrolyte was initially synthesized. Using two identical PA electrodes and the electrolyte, a symmetric split supercapacitor cell was built. Electrochemical measurements were conducted in a voltage window from −0.5 to 0.5 V at a current density of 1 A g^−1^.

## 4 Conclusions

In summary, this work presents a simple method of bicontinuous microemulsion polymerization, which allows the synthesis of both PANI nanofibers and a 3D cross-linked architecture. Moreover, the capacitive behaviour of designed symmetric cells was also investigated utilizing CV and GCD tests of the above materials.

Based on the results, the PANI nanofibers obtained in the study show the best electrochemical properties among all synthesized materials.

FTIR confirmed the successful synthesis of PANI nanofibers. The morphology study also confirms that PANI nanofibers do indeed have a nanoform. PANI nanofibers have a specific capacitance of 280.4 F g^−1^ at a current density of 1 A g^−1^ and good cycling stability of 98% up to 1000 cycles. Moreover, this electroactive material showed good specific power and energy densities, equal to 900 W kg^−1^ and 14.02 W kg^−1^, respectively. These results are explained by and subject to its relatively large surface area and electrical conductivity.

On the other hand, it was found from the charge–discharge curves that 3D crosslinked PANI also tends to rapidly charge and discharge, which shows how quickly a supercapacitor can store and release energy.

The bicontinuous microemulsion nanoreactor we developed easily fabricates unique nanostructures of polymers and their 3D crosslinked networks, which potentially improve the supercapacitive performance of the obtained materials.

## Data availability

The data supporting this article have been included as part of the ESI.†

## Conflicts of interest

There are no conflicts to declare.

## Supplementary Material

RA-015-D4RA07348G-s001
