# Supplementary material for: Enhanced electrochemical performance of a polyaniline-based supercapacitor by a bicontinuous microemulsion nanoreactor approach
Source: RSC Adv. 2025 Jan 14;15(2):1205–11. doi: 10.1039/d4ra07348g (PMC11729238; doi:10.1039/d4ra07348g)
Supplement: RA-015-D4RA07348G-s001 [file RA-015-D4RA07348G-s001.pdf]

Supplementary Information (SI) for RSC Advances.
This journal is © The Royal Society of Chemistry 2025

Supporting Information

Enhanced Electrochemical Performance of Polyaniline-based Supercapacitor by Bicontinuous Microemulsion Nanoreactor Approach

Yelriza Yeszhan[a], Sagydat Duisenbekov[a], Dana Kurmangalieva[a], Dana Kazhigitova[a,b], Perizat Askar[a], Yerbol Tileuberdi[c], Aishuak Konarov[a] , Salimgerey Adilov[d], Nurxat Nuraje[a,e]

Author Information

Affiliations

[a]Department of Chemical and Materials Engineering, School of Engineering & Digital Science, Nazarbayev University, Astana 010000, Kazakhstan
[b]Department of Chemistry and Chemical Technology, Al-Farabi Kazakh National University, Almaty 050040, Kazakhstan
[c]Institute of Natural Sciences and Geography, Abai Kazakh National Pedagogical University, 13, Dostyk ave., Almaty 050010, Kazakhstan
[d]Department of Chemistry, School of Sciences and Humanities, Nazarbayev University, Astana, 010000 Kazakhstan
[e]Lab of Renewable Energy, National Laboratory Astana, Nazarbayev University, Astana, 010000 Kazakhstan

*sadilov@nu.edu.kz(S. Adilov)

*nurxat.nuraje@nu.edu.kz(N. Nuraje)

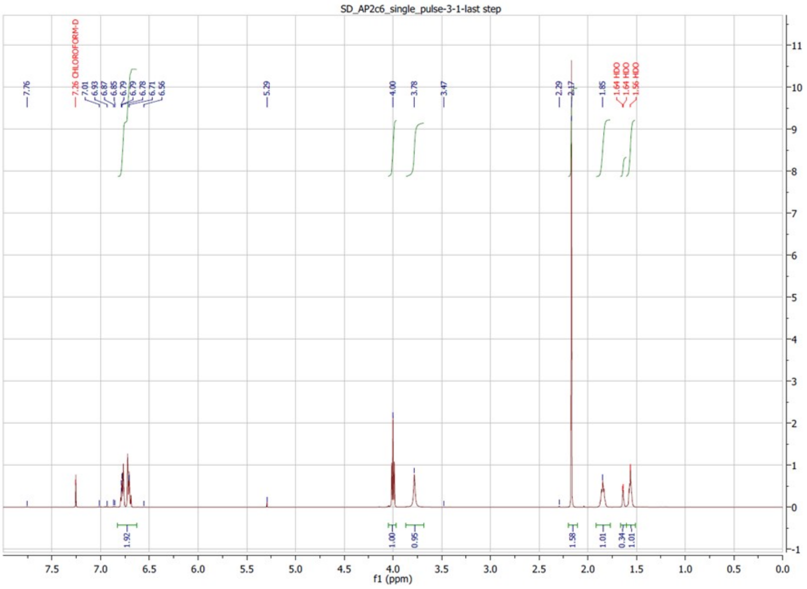

Figure S1. ^{1}H NMR of aniline linker compound (with acetone impurity)

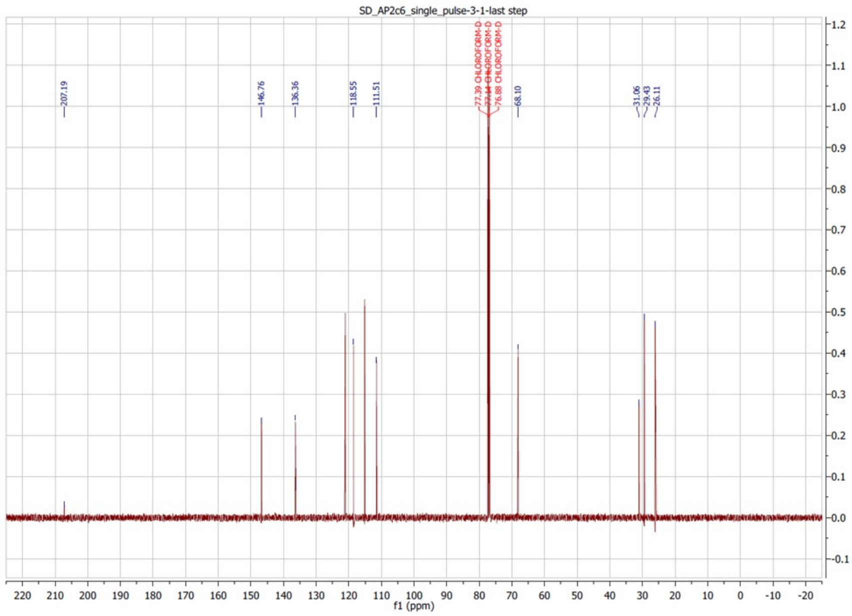

Figure S2. ^{13}C NMR of aniline linker compound (with acetone impurity)

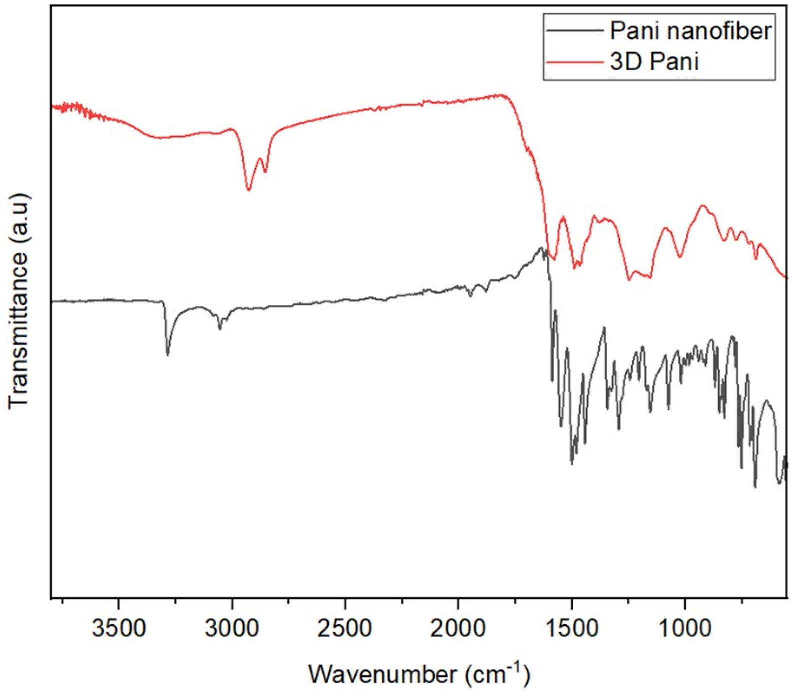

Figure S3. FTIR spectra of polymeric materials

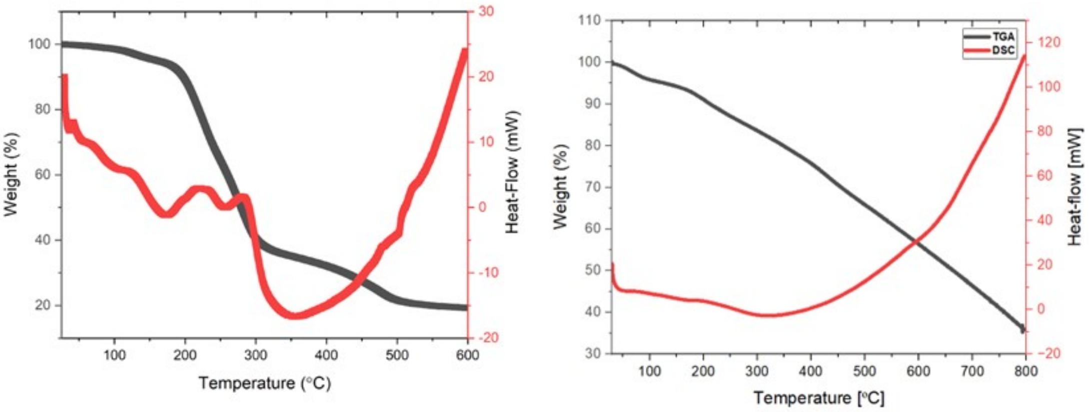

Figure S4. TGA/DSC of (a) PANI nanofiber and (b) crosslinked PANI

Figure S5 illustrates the BET N_2 absorption isotherms of the nanostructured PANI, 2D and 3D PANI. From the isotherm, can be observed that the amount of the adsorbed nitrogen is in this order 38.7 cm^3/g for PANI fiber > 30.74 cm^3/g for 3D PANI > 19.53 cm^3/g for 3D PANI-AB composite > 15.2 cm^3/g for 2D PANI film.

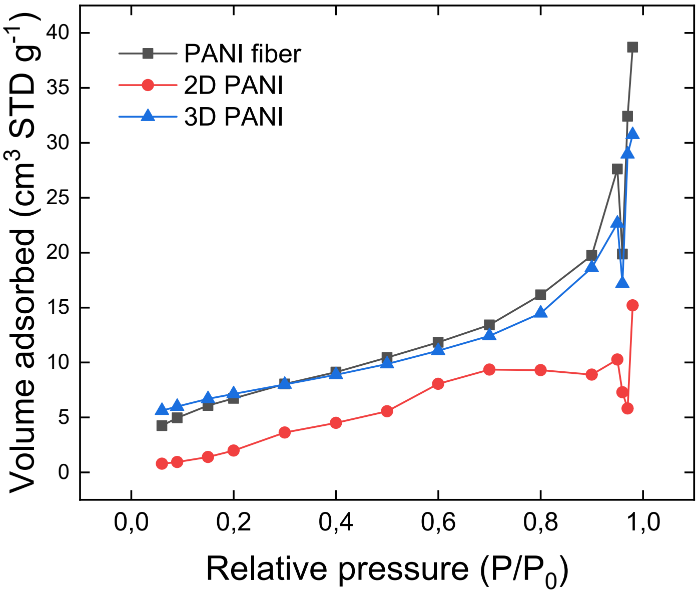

Figure S5. The nitrogen adsorption isotherms of polymeric materials
